# Targeted generation of complex temporal pulse profiles

**DOI:** 10.1038/s41598-022-07875-0

**Published:** 2022-03-09

**Authors:** Mariem Guesmi, Petra Veselá, Karel Žídek

**Affiliations:** Regional Center for Special Optics and Optoelectronic Systems (TOPTEC), Institute of Plasma Physics, Czech Academy of Science V.V.I., Za Slovankou 1782/3, 182 00 Prague 8, Czech Republic

**Keywords:** Ultrafast photonics, Ultrafast photonics

## Abstract

A targeted shaping of complex femtosecond pulse waveforms and their characterization is essential for many spectroscopic applications. A 4f pulse shaper combined with an advanced pulse characterization technique should, in the idealized case, serve this purpose for an arbitrary pulse shape. This is, however, violated in the real experiment by many imperfections and limitations. Although the complex waveform generation has been studied in-depth, the comparison of the effects of various experimental factors on the actual pulse shape has stayed out of focus so far. In this paper, we present an experimental study on the targeted generation and retrieval of complex pulses by using two commonly-used techniques: spatial-light-modulator (SLM)-based 4f pulse shaper and second-harmonic generation frequency-resolved optical gating (FROG) and cross-correlation FROG (XFROG). By combining FROG and XFROG traces, we analyze the pulses with SLM-adjusted complex random phases ranging from simple to very complex waveforms. We demonstrate that the combination of FROG and XFROG ensures highly consistent pulse retrieval, irrespective of the used retrieval algorithm. This enabled us to evaluate the role of various experimental factors on the agreement between the simulated and actual pulse shape. The factors included the SLM pixelation, SLM pixel crosstalk, finite laser focal spot in the pulse shaper, or interference fringes induced by the SLM. In particular, we observe that including the SLM pixelation and crosstalk effect significantly improved the pulse shaping simulation. We demonstrate that the complete simulation can faithfully reproduce the pulse shape. Nevertheless, even in this case, the intensity of individual peaks differs between the retrieved and simulated pulses, typically by 10–20% of the peak value, with the mean standard deviation of 5–9% of the maximum pulse intensity. We discuss the potential sources of remaining discrepancies between the theoretically expected and experimentally retrieved pulse.

## Introduction

Ultrafast laser pulses with a complex temporal structure are widely used in many application domains, including pulsed laser deposition^1^, optical code division multiple access^2,3^, and ultrafast spectroscopy^4–9^, femtochemistry^10,11^, or femtobiology^11^. In all these domains, the shaping of ultrafast optical waveforms is essential to develop their scientific and technological potential.

The targeted generation and characterization of complex pulses is seemingly a simple goal, which can be in the idealized case attained by the commonly used pulse-shaping and pulse-retrieval techniques^9,12–16^. However, the limitations imposed by the real-life experimental conditions make the targeted generation and subsequent retrieval of complex temporal waveforms a demanding task^17,18^.

The spectroscopic applications of pulse shaping frequently focused on the generation of a sequence of pulses, which are used in multidimensional electronic or vibrational spectroscopy^9,17,19^. Concerning the more general case, the article of Squier et al. studied the application of a complex phase on the pulses without evaluating the actual temporal shape of the attained pulses^20^. A distinct direction is the optical arbitrary waveform generation based on frequency combs, where the waveform is acquired by setting the phase line-by-line to the frequency modes^12–15^. However, such a pulse shaper can be only attained for a very high pulse repetition rate. This disqualifies their use in standard time-resolved spectroscopy of optical materials.

Overall, although complex pulses are commonly used in many spectroscopic experiments^5,6,9,10^, the role of various experimental limitations on the complex resulting pulse shape has stayed out of focus. Articles comparing the expected and the acquired complex pulse shapes typically suffer from noticeable discrepancies between the two waveforms, for which it is not clear whether the difference originates from the limitations of the pulse simulation or the pulse retrieval^14,21^. Since reliable control over the pulse shape is essential for many experiments, it is of high interest to identify the constraints of the targeted pulse shaping.

In this article, we study the link between the pulse waveform predicted by a pulse shaper simulation and the waveform attained from the experimental retrieval of the actual pulse. We combined one of the most commonly used techniques used in ultrafast spectroscopy—a 4f pulse shaper equipped with a spatial light modulator (SLM) for pulse shaping and second-harmonic generation (SHG) frequency-resolved optical gating (FROG) and cross-correlation FROG (XFROG) for the pulse characterization. Using pre-compressed 20-fs pulses, we generated waveforms ranging from simple pulse shapes to highly complex ones. By using a combination of two different characterization methods SHG–FROG and FROG–XFROG, we were able to achieve actual shapes of the pulses, which are compared then against the simulated pulses. For this comparison, we introduce a complex model, where the commonly known experimental limitations are taken into account. We study the effect of individual experimental factors on the resulting pulse shape.

To attain a reliable pulse retrieval, we used a simultaneous optimization of SHG–FROG and XFROG trace. These methods are intelligible, well studied, and inherently provide a consistency check to identify potential artifacts and experimental errors^22–25^. While the SHG–FROG experiment leads to the complex pulses to ambiguity in the pulse shapes, we show that the combined FROG–XFROG dataset provides a consistent pulse waveform irrespective of the used reconstruction algorithm.

Owing to the reliable retrieval of a complex waveform, we could compare the resulting reconstructed pulse shapes to those simulated by a simplified model of a 4f pulse shaper. First, we simulated a pulse shaper with idealized properties and subsequently for realistic behavior, where the commonly known experimental limitations are taken into account. Namely, we integrated into our calculations the limited SHG FROG bandwidth, SLM pixelation, finite focal spot size^26,27^, the SLM pixel crosstalk^28–30^, or Fabry–Perot-like interference fringes due to reflections on the SLM^31^.

Based on the results, we introduce a complex model, where we discuss the major factors governing the actual temporal pulse shape and suggest possible sources leading to discrepancies between the expected and retrieved waveform.

Our article also provides a guideline for the precision expected from the targeted pulse generation using the standard 4f pulse shaper.

### Simulation of pulse shaping

We have developed a pulse shaper model, which takes into account the most prominent and studied experimental effects present in pulse shaping, namely: phase pixelation in SLM, pixel crosstalk in SLM, a finite spot size of the focused laser beam, and interference fringes arising due to reflections on SLM-air interface.

The pulse shape *E*(*t*) can be determined through its spectral representation, since the knowledge of laser spectrum $S(\omega )$ and phase $\varphi (\omega )$ is sufficient to retrieve the pulse waveform via Fourier transform $\tilde{E}(\omega ) = {\mathcal{F}}\{ E(t)\}$. In the spectral domain, the complex representation of electrical field amplitude is given by:

1 $$\tilde{E}(\omega ) = \sqrt {S(\omega )} \exp (i\varphi (\omega )),$$

where $S(\omega )$ and $\varphi (\omega )$ are the spectral intensity and phase, respectively.

As an input of our model, we used the spectrum $S(\omega )$ and a phase $\varphi_{C} (\omega )$ of the pre-compressed laser pulse, i.e., input phase of the pre-compressed pulse measured without pulse shaper. The phase was corrected for the changes induced by pulse shaper without any SLM modulation using a Taylor series^32^.

2 $$\varphi_{in} (\omega ) = \varphi_{C} + L(\omega - \omega_{0} ) + \frac{1}{2}GDD \cdot (\omega - \omega_{0} )^{2} + \frac{1}{6}TOD \cdot (\omega - \omega_{0} )^{3} ,$$

where $\omega_{0}$ is the central frequency, *GDD* represents the group delay dispersion, and *TOD* is the 3rd-order dispersion. The second term *L* causes the pulse translation in time, which can be ignored in our calculations. At the same time, we neglected the higher dispersion terms, which did not have any prominent effect on the resulting pulse shape, as we discuss in the text below.

As the next step, we included the phase induced by the SLM, denoted as $\varphi_{SLM} (\lambda )$, which modifies the spectral phase of the incident pulse and it is responsible for the actual pulse shape. The SLM was calibrated before the measurement (see “Experimental and data processing procedures” section) to accurately describe the resulting pulse shape. Nevertheless, the desired $\varphi_{SLM}$ shape is distorted in the real experiment by the experimental restrictions, such as pixel discrete nature (pixelation), pixel crosstalk, or finite size of the modulated beam. Hence, the actual SLM phase $\varphi_{SLM} (\lambda )$ will be described as a convolution of the pixelized phase pattern set to SLM $\varphi_{set} (p(\lambda ))$ with a Gaussian point spread function (PSF):

3 $$\varphi_{SLM} (\lambda ) = { }\varphi_{set} (p(\lambda )) \otimes \frac{1}{{\sqrt {2\pi \sigma_{X}^{2} } }}{\text{exp}}\left( { - \frac{{p^{2} }}{{2\sigma_{X}^{2} }}} \right),$$

where *σ*_X_ is the standard deviation quantifying the pixel crosstalk, which can be extracted via an SLM calibration procedure^33^. As demonstrated in our recent work, the *σ*_X_ value equals 1 pix for the SLM used in our experiment^28^. The output phase of the desired pulse is given as the sum of the input unmodulated laser pulse $\varphi_{in} (\omega )$ and the actual phase induced by the pulse shaper $\varphi_{PS} (\lambda )$:

4 $$\varphi_{PS} (\lambda ) = \varphi_{in} (\omega ) + \varphi_{SLM} (\lambda ).$$

The spectrum of the laser $S(\lambda )$ can be calculated from the input laser spectrum by considering the interference fringes induced by the Fabry–Perot-like behavior of the SLM liquid crystals. Since the interference pattern depends on the set phase and pixel properties, we followed the procedure proposed by Wittenbecher et al. to evaluate this effect^31^.

By using Eq. (1), we can calculate the complex electric amplitude *E*_*PS*_ by using the corrected spectrum $S_{FP} (\lambda )$ and phase $\varphi_{PS} (\lambda )$. However, this is still not the resulting pulse spectral amplitude and phase. Due to the finite focal spot size of the beam in the 4f pulse shaper, the single spot on the SLM will not affect a single wavelength but rather a narrow range of the wavelengths set by the focal spot. Therefore, the actual electric field will be described as a convolution of the electric field of the pulse passing through SLM $E_{SLM} (\lambda )$ with a Gaussian PSF:

5 $$E_{out} (\lambda ) = { }E_{PS} (\lambda ) \otimes \frac{1}{{\sqrt {2\pi \sigma_{FC}^{2} } }}{\text{exp}}\left( { - \frac{{(\lambda - \lambda_{0} )^{2} }}{{2\sigma_{FC}^{2} }}} \right),$$

where *σ*_FC_ is the standard deviation, which is proportional to the spot size of the focused laser beam in the SLM plane. By measuring the cross-section of the focused laser spectrum, we found that *σ*_f_ is equal to 0.5 pix^28^. This convolution leads, among other effects, to prominent drops in laser spectra in the spectral region where the SLM-induced phase is abruptly changing.

By including all these effects, we calculated the expected pulse shape and the corresponding FROG trace, which can be compared with the experimental data. Therefore, we can quantify the pulse simulation quality based on the commonly used *G*-error. For the difference between the experimentally measured $I_{FROG}^{exp}$ and the simulated $I_{FROG}^{sim}$ FROG traces, we introduced the *G*-error definition analogously to the pulse retrieval:

6 $$G_{sim} = \sqrt {\frac{1}{{N^{2} }}\mathop \sum \limits_{\omega ,\tau } \left| {\left( {I_{FROG}^{exp} (\omega ,\tau ) - \mu \cdot I_{FROG}^{sim} (\omega_{i} ,\tau )} \right)} \right|^{2} .}$$

Both measured and simulated traces were normalized to their maximum value and the parameter $\mu$ was optimized to obtaining the minimum discrepancy.

## Experimental and data processing procedures

### Pulse shaping

**Figure 1:**
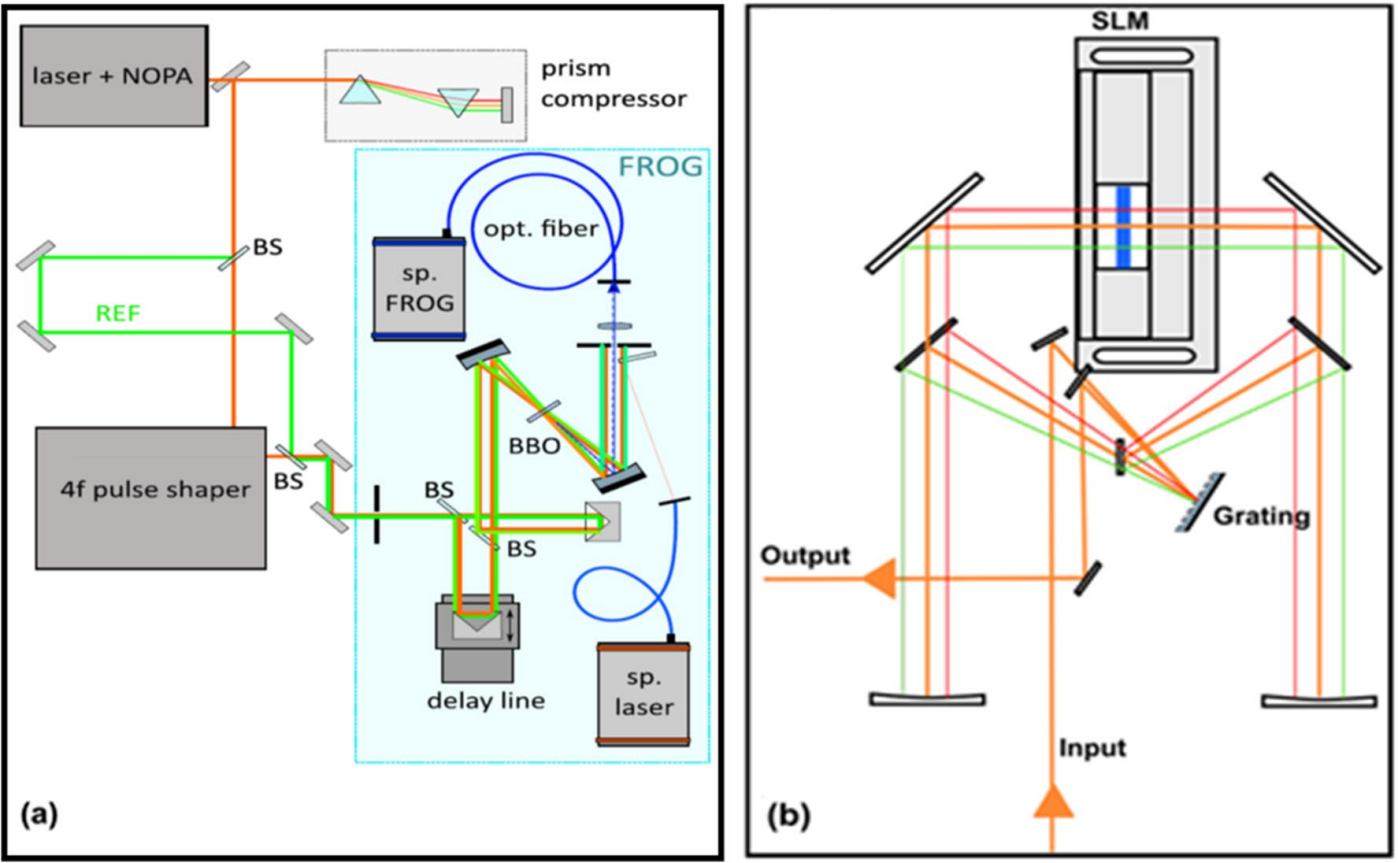
(**a**) The used experimental setup for the FROG (red beam only) and XFROG (both red and green beams are used). (**b**) The used pulse shaper in 4f geometry. See text for details of both setups.

The used experimental setup is depicted in Fig. 1. We employed an fs laser system Pharos (Light Conversion) operated at 1028 nm, which generated 290 fs long pulses at 10 kHz repetition rate, 100 µJ/pulse. A part of the output power (50 µJ/pulse) was converted by a non-collinear optical parametric amplifier (NOPA) N-3H Orpheus (Light Conversion) into a visible laser pulse at 640 nm, with the spectral width FWHM 757 cm^−1^. We used a prism pulse compressor integrated into the NOPA to adjust pulse length and to vary the dispersion. The pulse from NOPA was either directly characterized in FROG and XFROG setups, which are described later, or it was modified by a pulse shaper. We used a standard 4f pulse shaper—see Fig. 1b—employing grating 600 gr/mm, which spectrally disperse the beam in the horizontal direction. The dispersed beam was collimated along with the horizontal spectral shear and focused vertically by a spherical mirror (*f* = 500 mm) onto a spatial light modulator SLM-S640 (Jenoptik) placed in the Fourier plane. Consequently, symmetrically aligned mirrors were used to refocus the beam on the grating.

The pulse shaper was carefully calibrated by following a procedure of Döpke et al.^33^. First, we used the configuration, where SLM was placed between two crossed polarizers. The voltage variation on a single SLM pixel created a narrow peak in the laser spectrum, which enabled us to extract the wavelength corresponding to the pixel. Secondly, we measured a spectrogram of the light transmitted through the pulse shaper with a constant voltage applied on SLM. The varying intensity at each wavelength was recalculated into the phase shift introduced by the SLM. For this experiment, we kept SLM driving voltage within the range from 0.2 to 1.3 V.

Subsequently, we removed the crossed polarizers and measured the transmitted laser intensity modulation as a function of the applied voltage, following the procedure of Wittenbecher et al.^31^. Using this dataset, we considered the interference pattern induced by multiple reflections on the SLM interfaces. Finally, we also measured pixel crosstalk and focal spot size, following our previously published procedures^28^.

### FROG and XFROG

The temporal and spectral shape of the output pulse was measured by using the FROG and XFROG setups. In the FROG setup, the pulse was split with a pair of 50:50 beam splitters into two pulse replicas with an identical chirp. The delay between the pulses was varied by a delay line (PIMag Linear Stage). The pulses were focused on a 0.05 mm thick beta barium borate (BBO) crystal (Eksma Optics), where they generated a sum-frequency signal. Due to the non-collinearity of the incident pulses, the sum-frequency signal can be spatially separated by a pinhole. The transmitted SHG signal was coupled with a lens into a fiber and analyzed by a spectrometer (AvaSpec-ULS4096CL-EVO). The laser spectrum was measured from a beam reflection (Ocean Optics HR 2000)—see Fig. 1a.

The XFROG measurements were carried out with the same setup, yet with two different pulses. The reference (REF) pulse was split (10% intensity) before the pulse shaper and directed into the FROG experiment without passing through the pulse shaper. The test pulse passing through the pulse shaper and the pre-compressed REF pulse were measured by the FROG apparatus as a sequence of two pulses. I.e., the two replicas of this sequence of two pulses was created by the pair of 50:50 beam splitters. Care must be taken to set the timing between the test and the REF pulse so that their XFROG trace would not overlap with the FROG trace centered around the zero-time delay.

A variety of algorithms can be used to retrieve the pulse field from the FROG or XFROG trace. To reconstruct the pulse, we employed: ptychographic algorithm procedure by Sidorenko et al., COPRA procedure by Geib et al., IFT algorithm procedure by Trebino et al. and double-FROG algorithm procedure by Kida et al.^23,24,34,35^. To evaluate the quality of pulse retrieval, we employed the commonly used G-error^22,36^. If not stated otherwise, the data presented in the article were attained by the COPRA algorithm, and complete datasets for all algorithms can be found in the Supplementary Information.

Before the reconstruction, the experimental SHG FROG and XFROG traces were interpolated from the measured data to form ***N*** × ***N*** matrix to reconstruct ***N*** elements of intensity and phase vector. In all the presented reconstructions, we corrected the FROG traces for marginals^36^. To avoid the minor peripheral spectral regions, where the marginal correction would introduce high noise levels, we suppressed the regions where the FROG spectral marginal decreased well below 1% of its peak value. We included this limited bandwidth of SHG into our simulations, as we discuss in the respective section.

For the combined FROG–XFROG reconstruction, combined datasets were generated as 3*N* × *N* matrix. The central part 3*N* × *N* matrix was a superposition of normalized FROG traces of test pulse and REF pulse. The amplitude of the REF pulse FROG was adjusted to match fivefold lower REF pulse intensity; therefore, it has a very low intensity in the combined traces. Normalized XFROG trace (3*N* × *N* matrix) was used for the left part of the combined trace. Its amplitude was scaled to match fivefold lower REF pulse intensity. The same XFROG trace (3*N* × *N* matrix) flipped along the temporal axis was set as the right part of the combined trace.

In principle, the combined FROG–XFROG trace can be directly experimentally measured as a single dataset. This can be attained when the REF and test pulse delay is set close to zero yet high enough to avoid FROG and XFROG overlap. Nevertheless, this option is not versatile for the varying complexity of the test pulses. We tested this option experimentally, and we did not observe any significant improvement in the retrieval error by carrying out the reconstruction of the directly measured combined trace compared to the synthetic one.

## Results and discussion

The presented experimental work can be divided into two steps: (i) random pulse characterization and (ii) pulse simulation. The careful pulse characterization is a prerequisite for the second step. It allowed us to compare the simulated and actual pulse shapes and discuss whether the observed discrepancy is the result of imprecise pulse retrieval or approximations introduced in the pulse simulation.

### Random pulse reconstruction

**Figure 2:**
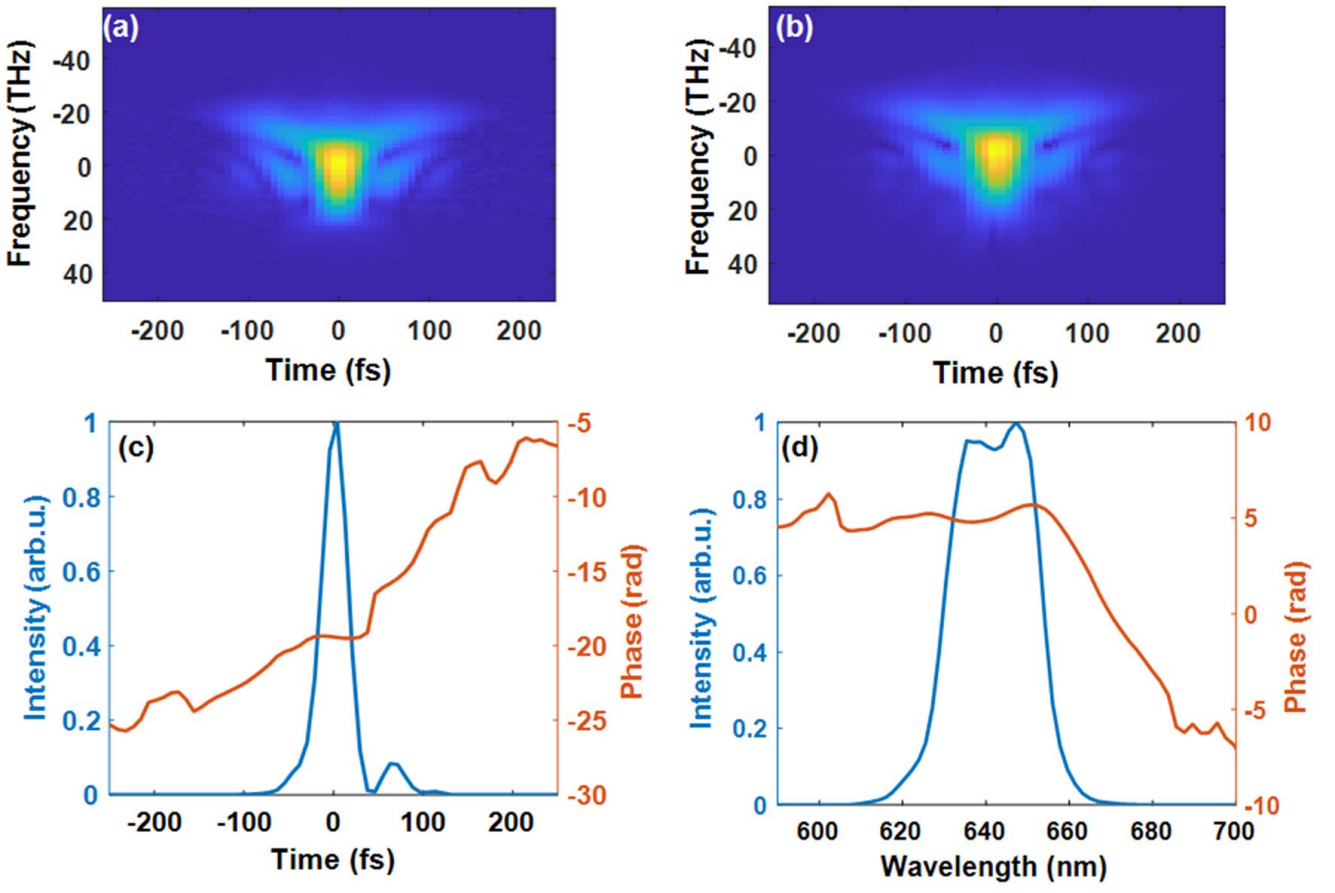
Measured (**a**) and retrieved (**b**) FROG traces (electric field amplitudes) from the input NOPA pulse. (**c**) Retrieved temporal intensity profiles and phases. (**d**) Retrieved spectral intensity profiles and phases. *N* = *96*, *T* = *400, dt* = *8.4211 fs*.

As the first step, we employed the SHG–FROG experiment to measure the shape of pre-compressed pulses generated by NOPA. The depicted experimental SHG FROG trace (upper left panel in Fig. 2) represents the electric field amplitude, i.e. $\sqrt {| {{\varvec{I}}_{{{\varvec{FROG}}}} ( {{\varvec{\omega}},{\varvec{\tau}}} )} |}$. This was reproduced by the reconstructed trace in Fig. 2b with a low *G* error value of 0.75%**.** The reconstruction provided us with the temporal and spectral intensities and phases of the pulse represented in the lower panels.

**Figure 3:**
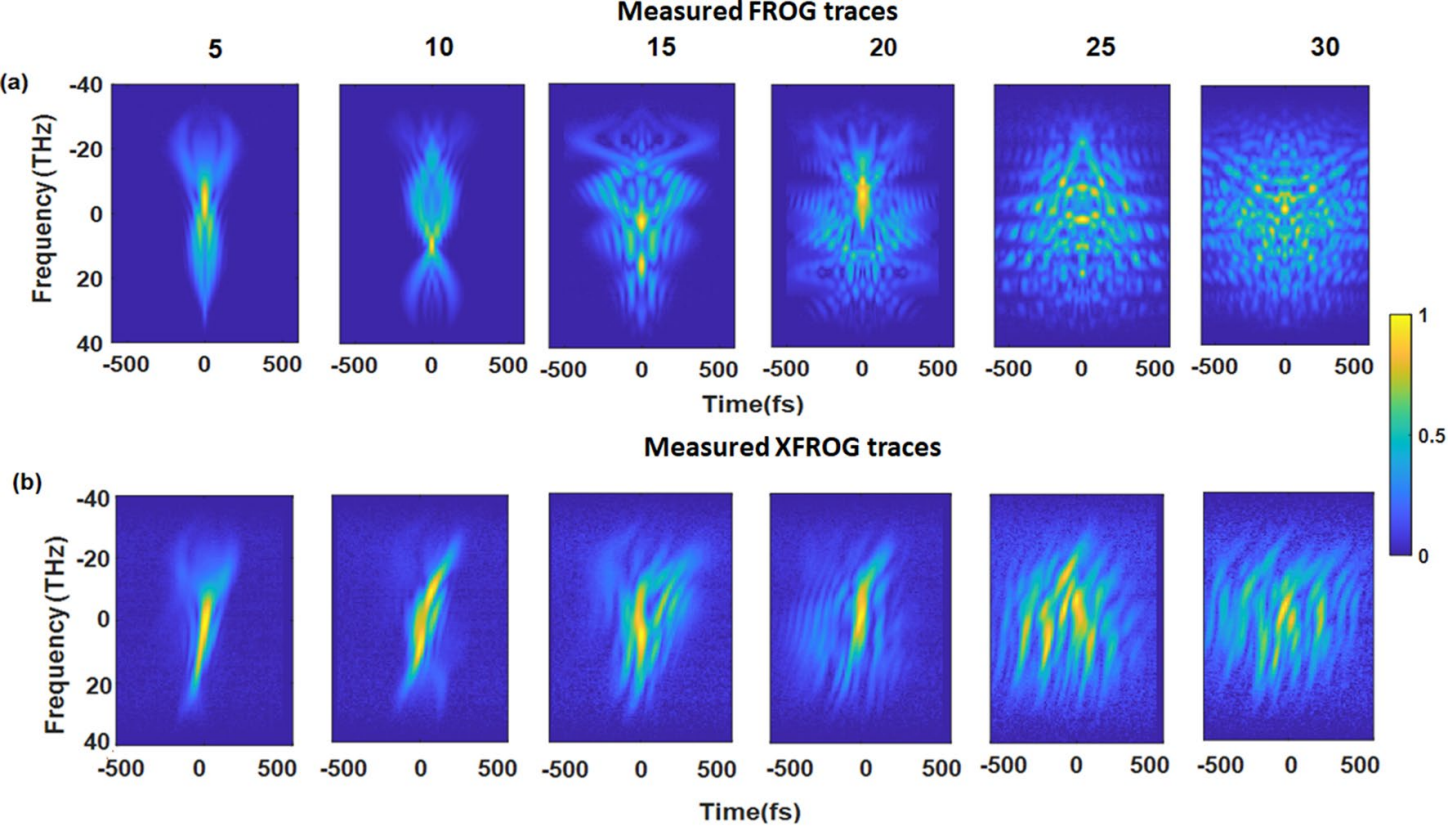
Normalized experimental (**a**) FROG and (**b**) XFROG traces (electric field amplitudes) of a pulse modified with a 4f-pulse shaper with SLM-adjusted phases. The traces were acquired for a set of random phases generated by the variation of the number of random guide points *N*_r_ from 5 to 30. *N* = *256, T* = *900, dt* = *7.06 fs.* Each trace was normalized to its maximum value.

The pre-compressed pulse was processed by a 4f-pulse shaper, where the phase was modulated with an SLM. We used a set of random phases generated by smooth spline interpolation of *N*_r_ equally spaced random numbers (0–2π) across the modulated spectral range (590–688 nm). The number of random guide points *N*_r_ was varied from 5 to 30, i.e., from less to more structured spectral phases. The corresponding FROG traces—see panels in Fig. 3a—and the XFROG traces—see Fig. 3b—were measured for each test pulse. As expected, for the increasing pulse complexity, i.e., increasing number of random points, the FROG and XFROG traces become more structured.

We used a REF pulse, which was split as a low-intensity replica of the NOPA pulse, to measure the XFROG traces presented in Fig. 3b. The REF pulse featured a pulse shape very similar to Fig. 2c, which was initially extracted from its FROG trace—see Fig. Sa in the Supplementary Information. Note that due to the relatively low intensity of the reference pulse, the experimental XFROG traces suffered a higher noise level than FROG. This becomes more prominent for the long pulses (high *N*_r_) as the total XFROG signal is smeared across a longer delay interval and its peak values are lower.

The most straightforward approach is to use the FROG trace itself to retrieve the pulse shape. According to the previous simulations, such reconstruction should be possible even for very complex pulses^20^. Therefore, we used the FROG traces depicted in Fig. 3a to extract the pulse shapes using three different algorithms: ptychographic algorithm^34^, COPRA^35^, and IFT algorithm^23,37^.

**Figure 4:**
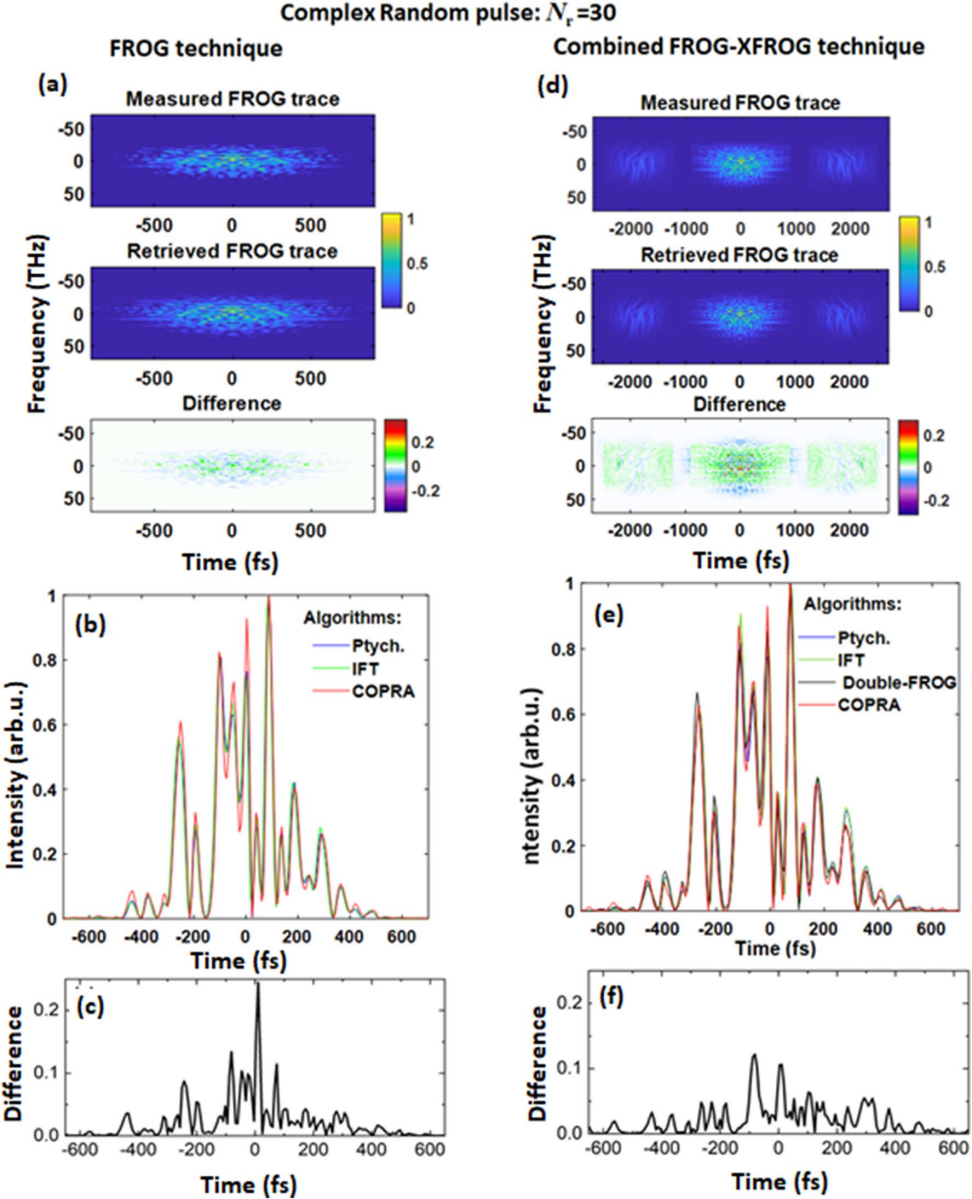
(**a**,**d**) Measured and retrieved FROG trace (electric field amplitude) of a complex random pulse: *N*_*r*_ = 30, and the difference of the measured and retrieved traces. (**a**) FROG technique: the trace was retrieved using Eq. (1), where *N* = *256, T* = *900, dt* = *7.06 fs*. (**d**) Combined FROG–XFROG technique: The trace was retrieved as a FROG trace of a double pulse, where *N* = *256, T* = *900, dt* = *7.06 fs*. (**b**,**e**) Retrieved temporal intensity profiles using three different algorithms for FROG (**b**) and Combined FROG–XFROG (**e**) techniques. (**c**,**f**) Difference between the maximum and the minimum of the temporal intensity profiles for the different algorithms for FROG (**c**) and Combined FROG–XFROG (**f**) techniques.

In Fig. 4a, we illustrate the FROG retrieval via COPRA algorithm for the most complex test pulse (*N*_r_ = 30)—see Supplementary Information Figs. S2 and S3 for all reconstruction algorithms. Using the three different algorithms applied to the experimental FROG trace, we attained pulses with very similar shapes. Nevertheless, the pulse waveforms differed in the intensity of the respective features—see, for instance, peaks between the times of − 200 fs and 0 fs in Fig. 4b.

Besides the FROG and XFROG techniques, their combination has been used to improve the robustness of the pulse reconstruction and recover both test and reference pulses simultaneously. This is possible with the double-blind FROG technique^37,38^ or by measuring a sequence of two pulses at a different wavelength using a standard FROG setup^24^.

Therefore, we combined the FROG and the XFROG (see Fig. 4d) by merging the FROG traces of the test pulse, the REF pulse, and the XFROG trace into a single combined dataset—see the “Experimental and data processing procedures” section for details. The combination provided us with an SHG FROG trace corresponding to a sequence of two pulses—REF and test pulse. Hence, we could simultaneously optimize both merged REF and test pulses by using the same three algorithms as for the test pulse FROG only. As an initial guess, we used the pulse shapes determined by their FROG traces. Another approach, which was published as the double-FROG algorithm by Kida et al.^24^, was also used to optimize these pulses iteratively.

For the combined FROG–XFROG, we observed that all pulse retrieval methods reliably converged into a consistent pulse shape with subtle differences between the retrieved curves even by employing various reconstruction algorithms—see Fig. 4b,e. This became apparent also when we evaluated the difference between various retrieved waveforms. We calculated the difference between maximum and minimum retrieved pulse intensity for each time—see Fig. 4c–f. For the combined FROG–XFROG retrieval, this difference decreased to approximately 50% of the values from only FROG retrieval. Nevertheless, the FROG method itself is sufficient for less complex pulses, especially when the overall pulse shape is the information of interest.

**Figure 5:**
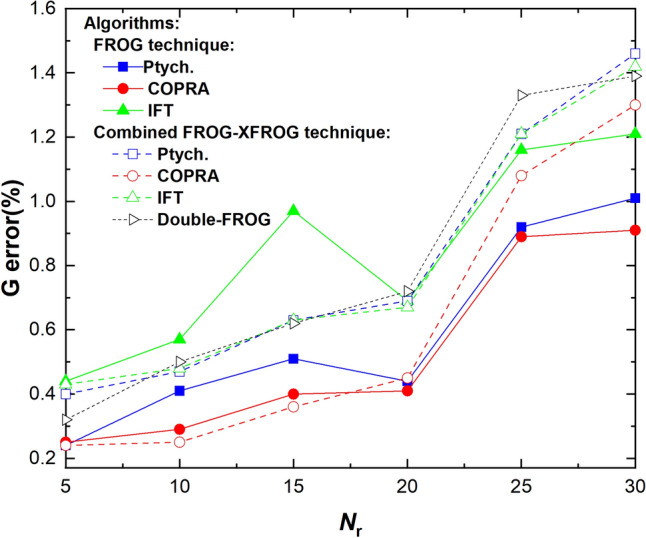
*G* error versus the number of random phase guide points *N*_r_ from 5 to 30, for the FROG (solid lines, solid symbols) and the combined FROG–XFROG techniques (dashed lines, open symbols) using different algorithms: Ptychographic, COPRA, IFT, and Double-FROG. The lines serve only as a guide to the eye.

To evaluate the used algorithms quantitatively, we studied the *G* error between the measured and the retrieved FROG traces for each number of the random guiding points *N*_r_—see Fig. 5. We found that the COPRA algorithm provided lower *G* error values for both FROG and FROG–XFROG datasets. Therefore, we used the pulse shapes retrieved by COPRA from the combined FROG as the actual pulse shape generated by the pulse shaping. Nevertheless, as we showed in Fig. 4 (b), the higher *G*-error for other methods corresponds to very subtle changes in the pulse shape.

Overall, the *G* error was very low for mildly modulated spectra with *N*_r_ up to 20. We ascribe the sudden increase for *N*_r_ = 25 to the limited spectral resolution of the spectrometer collecting the FROG trace spectra, which features 0.6 nm resolution at 300 nm. The rapid modulation of the spectral phase (around 3 nm-wide features for *N*_r_ = 30) leads to sharp spectral features in the FROG trace. The features were not fully captured by the spectrometer, and the retrieval-experiment agreement can be consequently diminished.

### Random pulse simulation

By using the combined FROG–XFROG trace, we were able to extract with high precision the actual pulse shape generated by the pulse shaper for all the used SLM phases. This allowed us to link the resulting pulse shape, which served as ground truth, with the expected shape induced by the pulse shaper itself.

We simulated the input NOPA pulse after its propagation through the SLM. As a starting point, we used the laser spectrum of NOPA pulse, which was restricted numerically by multiplying the laser spectrum with the Gaussian function to account for the limited conversion bandwidth of the used BBO crystal in the FROG setup. This approach is not fully equivalent to the actual phase-matching restrictions set by the SFG interaction. However, it is a reasonable approximation in our experiment since the laser spectrum changes were minor—see Supplementary Information for more details.

Based on the calculations of phase-matching conditions, the bandwidth was set to 55 nm FWHM, which was in good agreement with the observed FROG marginal. The spectrum served in the simulations as $S(\omega )$ in Eq. (1).

In the idealized case, we can simply add the smooth spectral phase set on the SLM pixels and calculate the pulse shape. In other words, we would ignore the effect of pixelation, pixel crosstalk, and finite focal spot. Minor GDD and TOD correction of 50 fs^2^/mm and 10 fs^3^/mm, respectively, were attained by fitting via minimization of the total *G*_sim_ value between the measured and the simulated NOPA FROG trace.

**Figure 6:**
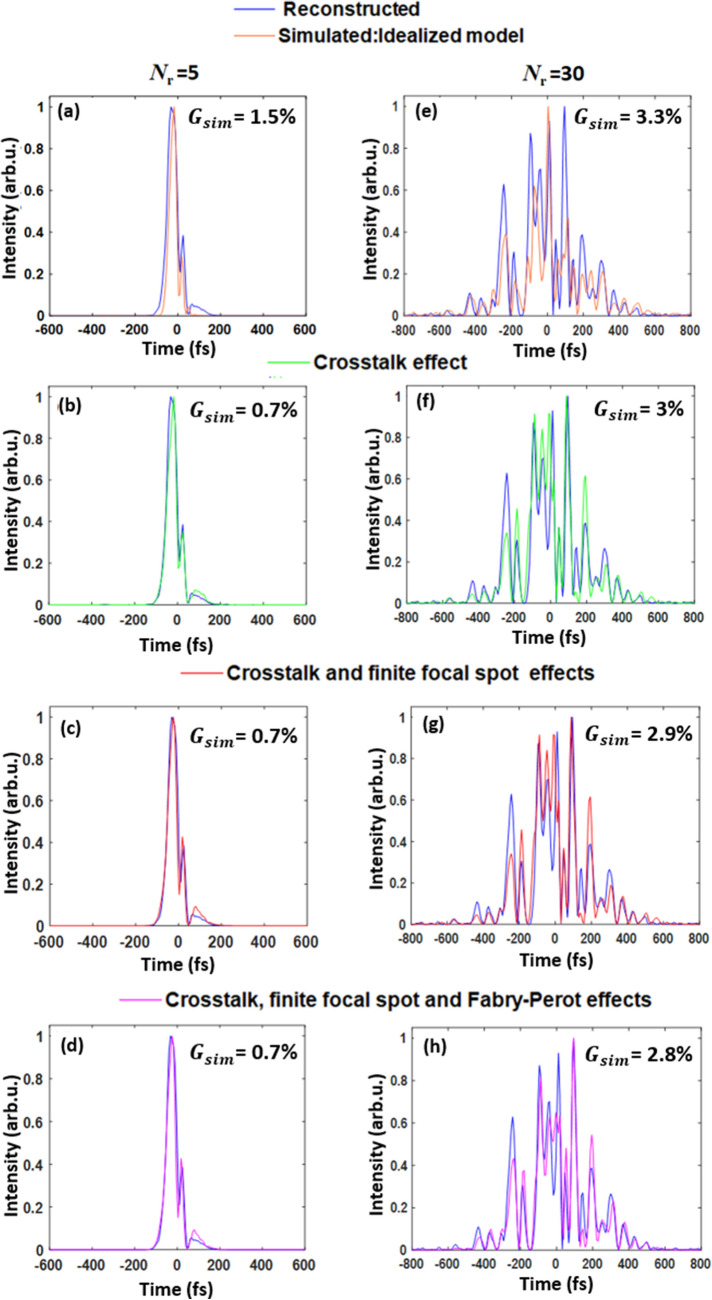
Retrieved (blue lines) and simulated temporal intensity profiles of pulses with SLM-adjusted spectral phase: *N*_*r*_ = 5 (**a**–**d**) and *N*_*r*_ = 30 (**e**–**h**). Simulated pulses: (**a**,**e**) idealized model; (**b**,**f**) SLM pixel crosstalk effect; (**c**,**g**) SLM pixel crosstalk and finite focal spot; (**d**,**h**) SLM pixel crosstalk, finite focal spot, and SLM-induced interference.

Idealized pulse shapes are depicted in Fig. 6a,e as orange lines. We compare the situation for the simplest and the most complex studied pulse shown in the left and right panels, respectively. We can spot a good agreement between the simulated and retrieved pulse for the simple pulse shape with a relatively low *G*-error value between the measured and the simulated data *G*_*sim*_ = 1.5%. On the contrary, the shape of the complex pulse is clearly different—see Fig. 6e with a high *G*-error (*G*_*sim*_ = 3.3%).

As the next step, we included the effect of the phase pixelation and pixel crosstalk in SLM, which caused the actual set phase to be step-like and subsequently smeared across the pixels. These were described as a convolution of the pixelized phase set to SLM with a Gaussian PSF using Eq. (3), where the standard deviation *σ*_X_ is equal to 1 pix—see Fig. S4 in the Supplementary Information for the data. For the simple pulses (Fig. 6b), we observe that the temporal intensity profile reaches a nearly ideal shape with this correction. The G-error is highly improved to 0.7%.

When we focused on the more complex pulse (*N*_*r*_ = 30) (Fig. 6f), we observed that the pulse shape is highly altered by each phenomenon included in the simulation. Nevertheless, the improved simulation-experiment agreement can be observed on the G-error value. The SLM pixelation and pixel crosstalk reduced the *G*_*sim*_ error from 3.3% (idealized case) to 3% (SLM properties included). The effect of the finite focal spot expressed by Eq. (5) and depicted in Fig. 6c,g as red lines reduced the *G*_*sim*_ error to 2.9%—see Fig. 6g. Finally, by including the Fabry–Perot-like interference fringes in spectra, as represented in Fig. 6d,h as pink lines, the *G*_*sim*_ error reached 2.8%—see Fig. 6h. While the *G*_*sim*_ error is only partly reduced by including all the studied phenomena, we obtained a highly improved agreement between the retrieved and experimental pulse waveforms.

**Figure 7:**
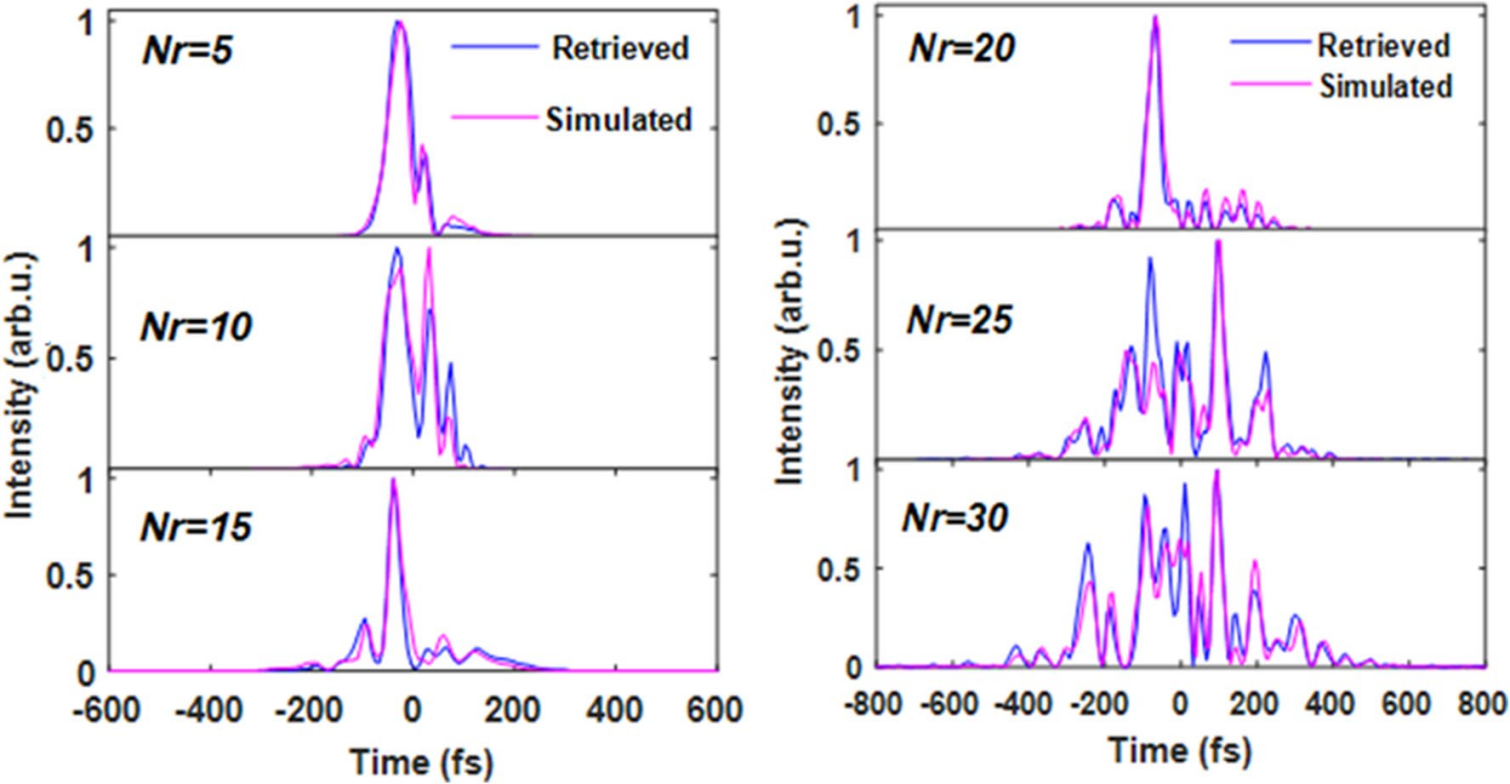
Experimentally retrieved (blue lines) and simulated (magenta lines) temporal intensity profiles of pulses processed with a 4f-pulse shaper with SLM-adjusted random phases *N*_*r*_ from 5 to 30 (see text for details). The pulses were simulated by the complete model.

Figure 7 provides an overview of the simulated and retrieved waveforms for all *N*_r_. For some of the studied SLM phases, we reached an excellent agreement—namely, for the random phases generated with the number of random guides *N*_*r*_ = 5 and 20. However, severe discrepancies between the simulated and measured shapes can be spotted even by including the realistic model—see the pulses with *N*_*r*_ = 10 or 25. The difference between the normalized simulated and retrieved waveforms reaching up to 20% can be attributed to the uncertainty in the pulse retrieval, as is apparent from Fig. 4f. However, the actual difference between some of the curves in Fig. 7 clearly exceeds these values.

Since many model parameters were measured experimentally, they suffered from uncertainty. They could contribute to the differences in the temporal shape observed in Fig. 7. To exclude the possibility that the differences are the result of the imprecise parameters, we created a complex multi-step fitting procedure described in detail in Supplementary Information. The model parameters, including, for instance, SLM pixel crosstalk σ_CT_, were left free to iteratively attain the best agreement between the six retrieved and simulated waveforms. If the uncertainty in the model parameters was the main cause of the observed differences, the free fitting would highly improve the simulation—experiment agreement. Nevertheless, the free fit of the simulation parameter for our data did not lead to a considerably better agreement between the simulated and retrieved pulses.

Therefore, we can securely conclude that the discrepancy between the simulated and retrieved pulses in Fig. 7 is not a result of imprecise parameters or calibration steps of our model. Instead, this difference could only be explained by factors, which were not fully covered by the simulation. Firstly, the used numerical bandwidth reduction does not fully correspond to the phase-matching conditions set by the SFG process in the FROG experiment. However, in our experiment, the difference in the original and bandwidth-reduced spectrum was minor (see Supplementary Information). Thus, the contribution of the spectral “wings” to the FROG signal was negligible.

Secondly, we can speculate that the actual SLM crosstalk effect follows a more complex SLM-voltage-dependent model than the simple convolution introduced by Eq. (3). Finally, some of the parameters, such as focal spot size, will differ across the beam due to the varying wavelength and aberrations in the pulse shaper. It is also worth mentioning that we assumed in our simulation an idealized case, where the pulse waveform is the same across the beam^26,27^.

## Conclusion

In conclusion, we presented a combination of FROG and XFROG techniques applied to characterize complex random pulses. The pulses were generated from a NOPA and processed by a 4f-pulse shaper via an SLM-adjusted spectral phase. We used a set of random phases, ranging from relatively simple waveform to highly structured pulses.

We demonstrated that the FROG technique itself is sufficient to characterize a complex pulse. Yet, the combined FROG–XFROG is the more reliable and reaches highly consistent waveforms independently of the used retrieval algorithm. Nevertheless, even this combination introduces uncertainty, locally reaching 10–20% of the pulse peak value.

Subsequently, we compared the retrieved pulses with a simplified model describing the behavior of an SLM-equipped 4f pulse shaper. While the simplest waveforms could be reproduced with a reasonable precision even by a simple idealized model, this model failed to provide a high fidelity for the complex phase spectral profiles. Therefore, we included in our model also the effect of SLM pixelation, SLM pixel crosstalk, finite focal spot of the beam, and interference fringes induced by SLM. Each of the steps improved the overall agreement between the simulated and retrieved pulses.

As a result, we attained a very good agreement of the simulated and retrieved waveforms, where the difference between the pulse shapes consisted only in the relative weight of various peaks. Nevertheless, we can clearly spot—analogously to previous publications on pulse shaping^13,14^—that certain features in spectra differed by more than 30% of the peak values. Such discrepancy cannot be simply assigned to the uncertainty of the pulse retrieval. By fitting many parameters of the model, we checked that the discrepancy between the simulated and retrieved pulses cannot originate from imprecise calibration steps. This suggests that the origin of the discrepancy is the approximations used in our model. The nature of these will be addressed in our future research. Nevertheless, we can speculate that the model uses an oversimplified description of the SLM behavior, assumes a constant focal spot for all wavelengths, or we need to take into account the space–time coupling induced by the finite focal spot.

Currently, the presented model, which takes into account the finite SHG bandwidth, SLM pixelation, SLM pixel crosstalk, finite focal spot, and SLM-induced interference fringes, provides the precision, where the difference between the simulated and retrieved pulse can reach the standard deviation of 3–9% of the pulse intensity peak.

## Supplementary Information

Supplementary Information.
