# Supplementary material for: Targeted generation of complex temporal pulse profiles
Source: Sci Rep. 2022 Mar 9;12:3827. doi: 10.1038/s41598-022-07875-0 (PMC8907224; doi:10.1038/s41598-022-07875-0)
Supplement: Supplementary file 1 — Supplementary Information. [file 41598_2022_7875_MOESM1_ESM.pdf]

Targeted generation of complex temporal pulse profiles: supplemental document

Mariem Guesmi, Petra Vesela and Karel Žídek

Regional Center for Special Optics and Optoelectronic Systems (TOPTEC), Institute of Plasma Physics, Czech Academy of Science v.v.i., Za Slovankou 1782/3, 182 00 Prague 8, Czech Republic

Random pulse reconstruction

We used two different characterization techniques SHG-FROG and combined FROG-XFROG to retrieve the complex pulse shapes. For each technique, we applied various retrieval algorithms: ptychographic (hereafter abbreviated as "ptycho"), COPRA, IFT, and Double-FROG algorithm.

We used the number of iterations as the stopping criterion (ptycho, IFT, and double-FROG algorithm – 200 iterations; COPRA – 1000 iterations). The number of iterations was set high enough so that the algorithm always converged to a particular solution, and the objective function value did not decrease with the iterations. Although such an approach is not time-wise economical (typical reconstruction time was about 15 minutes for the combined FROG-XFROG traces). Nevertheless, the main current limitation is the acquisition time of the stretched FROG and XFROG trace. Therefore, we have not focused on the speed of the pulse retrieval procedure, which can be highly optimized.

In our reconstructions, we initially tested the pulse retrieval with a high number of runs, where each run used a different initial random phase. According to our observations, the correct pulse retrieval converged to the same pulse shape, and G error accounted for approx. 90% of the runs. In the remaining approx. 10% cases, the retrieved trace suffered from a very high G error. Differences between the correct individual runs of each algorithm were significantly lower than the differences between the algorithms themselves.

Therefore, as a general practice, we carried out the pulse retrieval procedure for 3 independent runs. This immensely reduces the possibility of attaining a misleading reconstruction (about 0.1% probability), and the potential flaw can be readily identified by a suspiciously high G error value.

Reference pulse

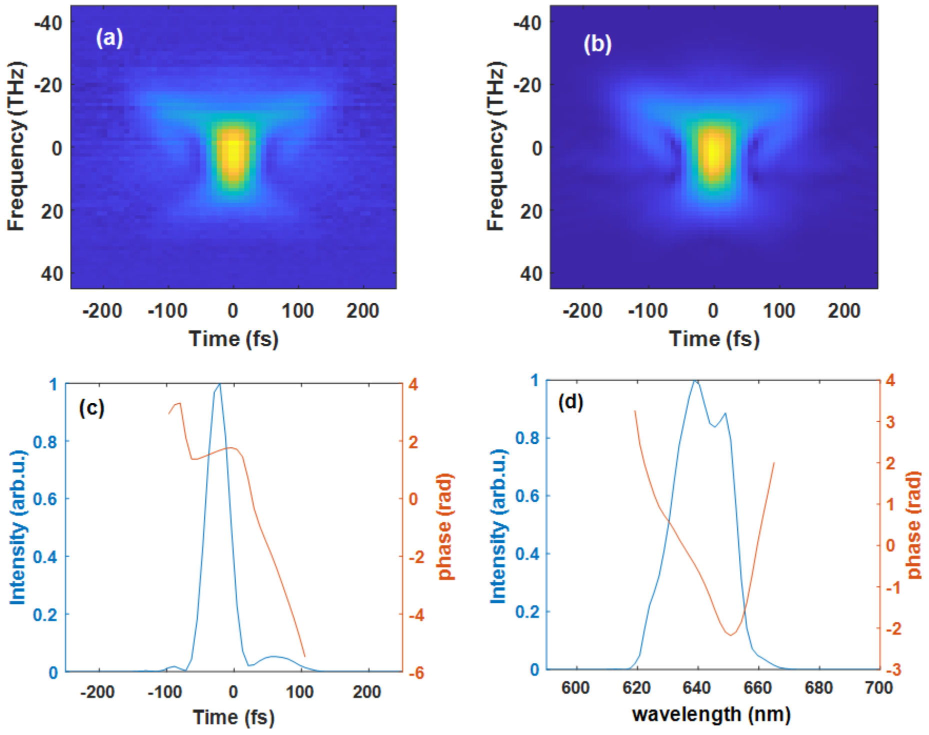

Figure S1. Measured (a) and Retrieved (b) FROG traces from a reference pulse. (c) Retrieved temporal intensity profiles and phases. (c, d) Retrieved spectral intensity profiles and phases. *N=96, T=400, dt=8.4211fs.*

FROG technique

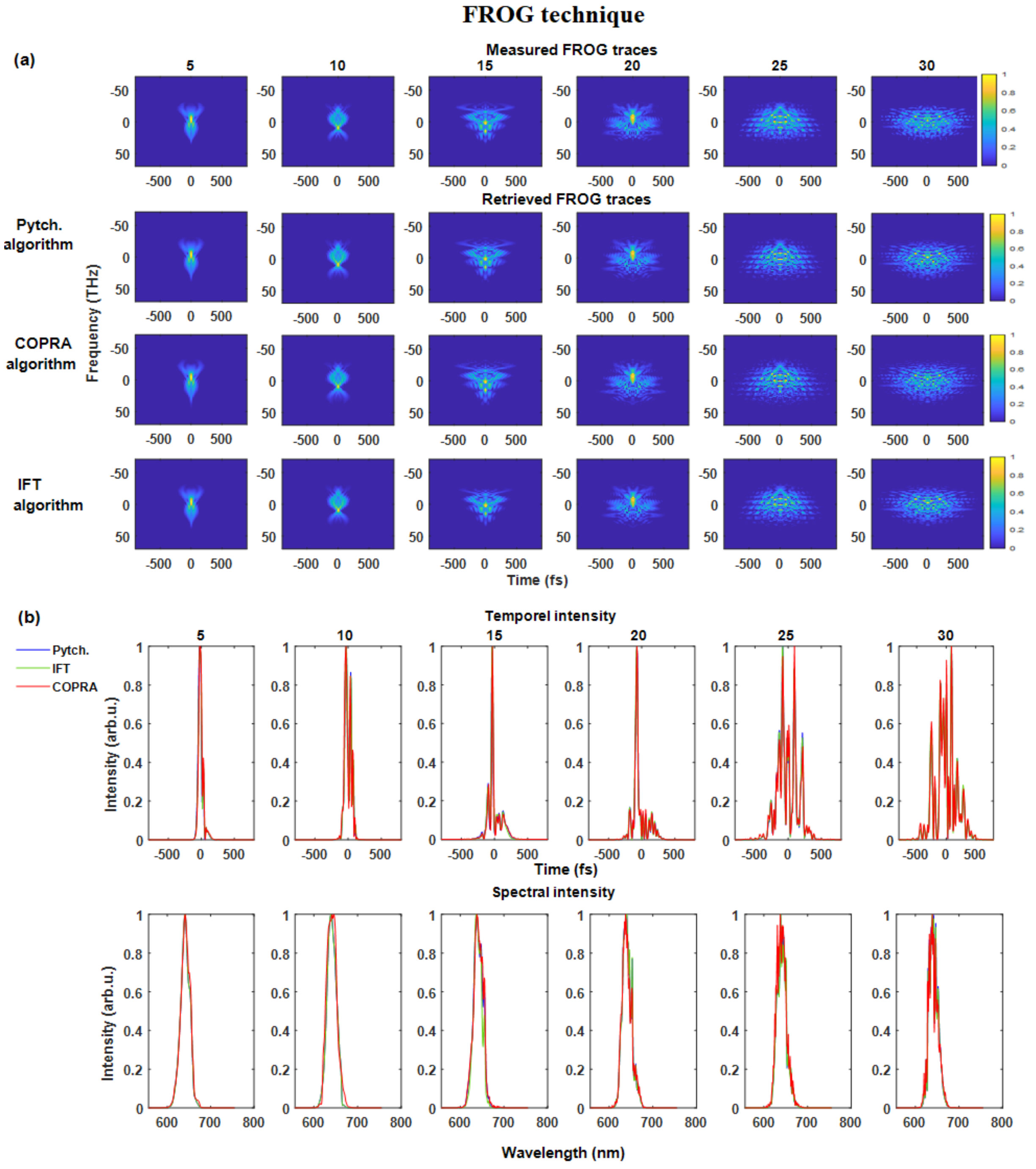

Figure S2. (a) Measured and retrieved of pulses, using three different algorithms (ptychographic, COPRA, and IFT), generated from NOPA and measured with a 4f-pulse shaper with SLM- adjusted phases (The random phases were generated by variation of the number of random guide points Nr from 5 to 30). (b) Retrieved temporal and spectral intensity profiles, respectively. The trace was retrieved using Eq. (1).

Combined FROG-XFROG technique

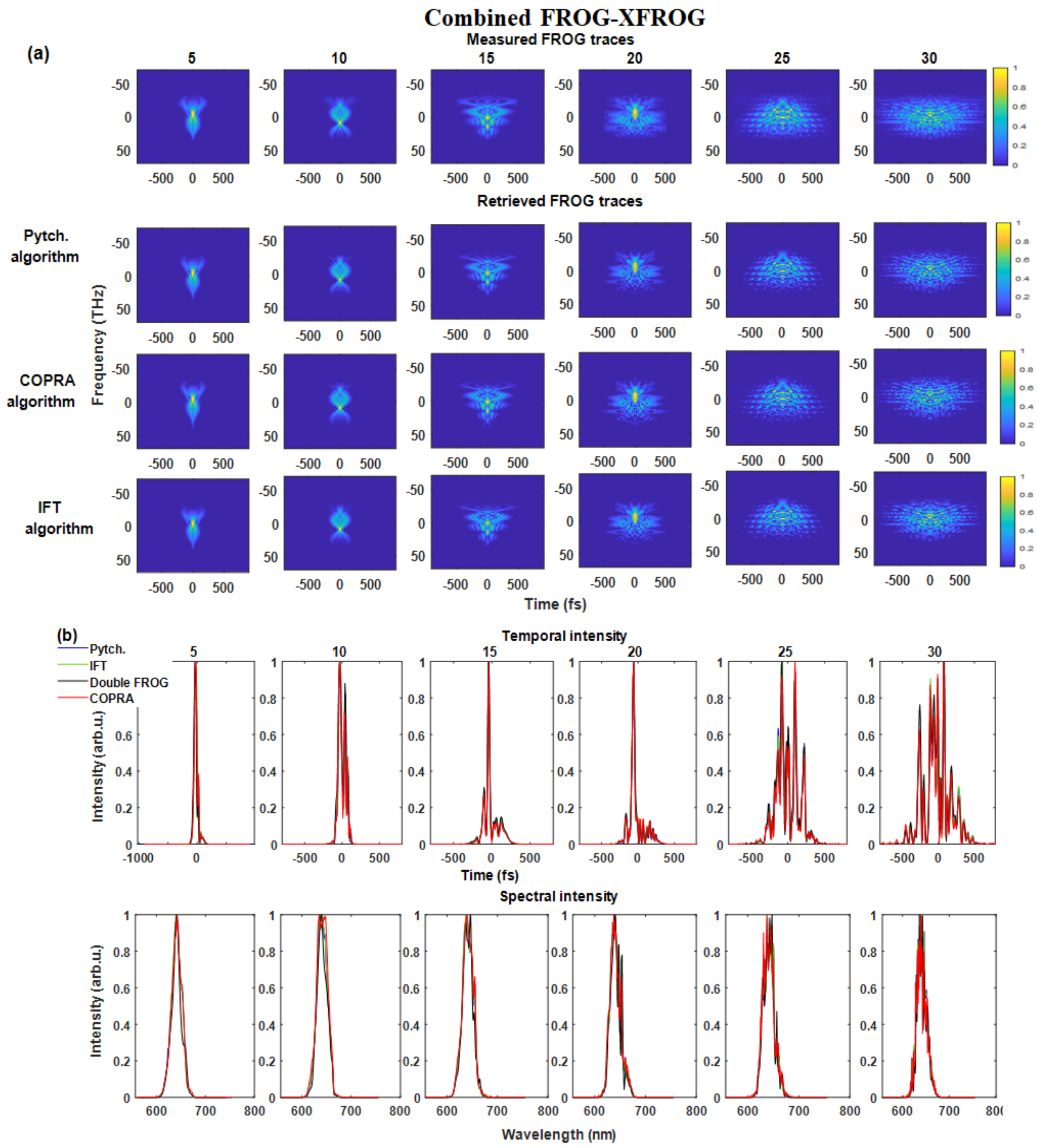

Figure S3. (a) Measured and retrieved of pulses, using different algorithms (ptychographic, COPRA, and IFT), generated from NOPA and measured with a 4f-pulse shaper with SLM- adjusted phases (The random phases were generated by variation of the number of random guide points Nr from 5 to 30). (b) Retrieved temporal and spectral intensity profiles, respectively. The trace was retrieved using Eqs. (2).

Applied phase modulation

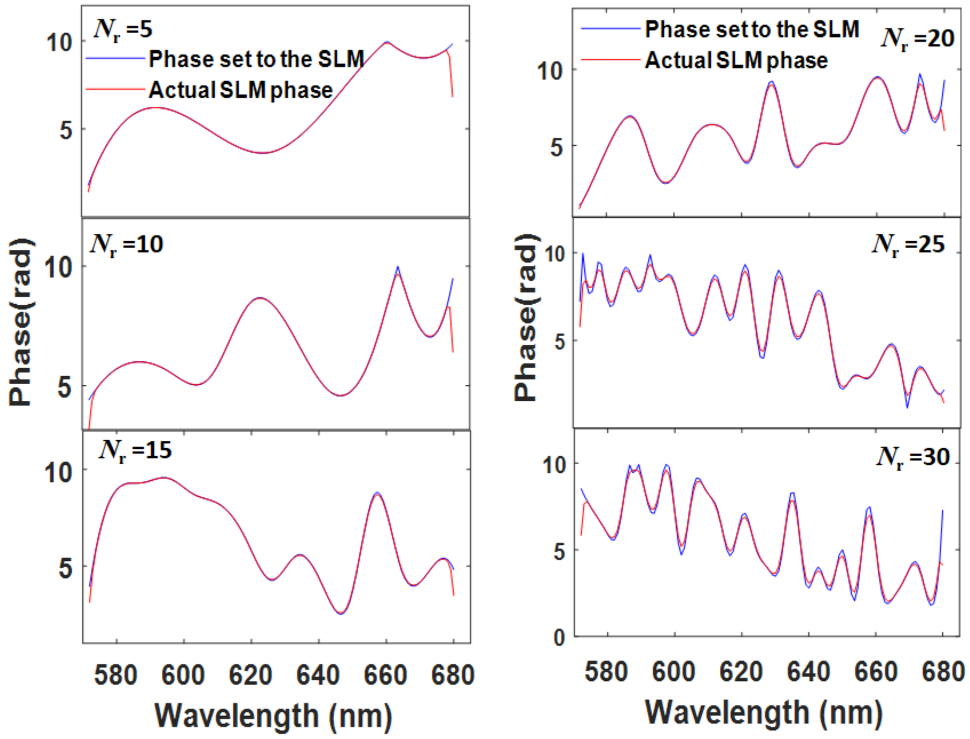

Figure S4. Actual SLM phase (red line), which is described as a convolution of the pixelized phase pattern set to SLM (blue line) with a Gaussian PSF. The standard deviation of the Gaussian is equal to 1 pix. Note that the decreased phase value at the axis edge does not affect the results since the laser spectrum is equal to zero in this region.

Laser bandwidth reduction

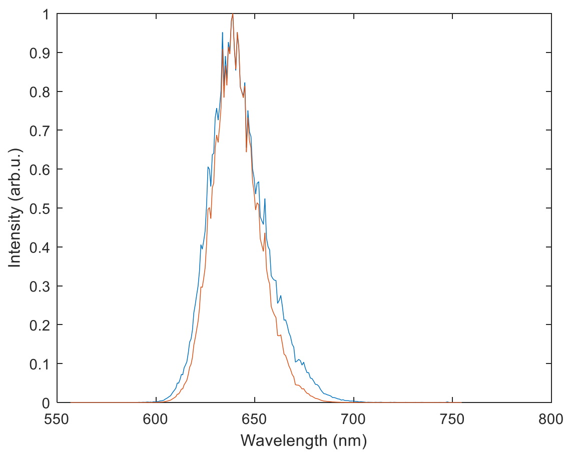

Figure S5. Example of laser spectrum used for pulse simulation before the correction (blue line) and after the correction (red line). Laser spectrum was generated from NOPA and processed with a 4f-pulse shaper with SLM- adjusted phases (N_r = 30).

Global fitting procedure of model parameters

The observed discrepancies between the experimental pulse shapes and simulated pulse shapes might arise due to the inaccuracy of the model parameters. Therefore, we created a complex multi-step fitting procedure. The model parameters were freely fitted to attain the best experiment—simulation agreement.

We identified the potential sources of inaccuracy, which included: (i) shift in the wavelength calibration, (ii) phase calibration shape, (iii) higher-order dispersion terms in Eq. (2), (iv) pixel crosstalk σ_{CT} value, (v) focal spot size in pulse shaper, (vi) laser spectrum shape and bandwidth. The optimized merit function was the overall agreement between the six retrieved and simulated waveforms, which was minimized for various parameter combinations. Subsequently, we simultaneously fitted all the parameters from a reasonable initial guess.

As stated in the discussion of Figure 7, we carried out extensive fitting trials to improve the agreement between the simulated and retrieved pulse waveforms. As the minimized merit function *M*, we used the total difference between the simulated pulse intensity waveform I_{sim} and experimentally retrieved intensity waveform I_{exp} for each pulse *i* and time *t*:

$$M = \sum_{i,t} \left(I_{sim}^{i}(t) - \mu^{i}.I_{exp}^{i}(t)\right)^{2}$$

The linear spectral phase of the simulated pulse, i.e., its shift in time and coefficient μ were adjusted to get the most optimum agreement with the simulated pulse. This merit function was selected because the primary goal of our effort was to reliably reproduce the temporal intensity profile of the pulses. Since I_{sim} is calculated from our model and depends on many parameters, we fitted the merit function for a variety of parameters:

1. The overall phase of the NOPA pulse (see Eq. (2)): GDD, TOD, fourth- and fifth-order dispersion value.
2. The shift in the spectral correction – see Eq. (3): $p_c(\lambda) = p(\lambda) + \Delta p$
3. Pixel crosstalk degree σ_X – see Eq. (3)
4. Laser beam focal spot size σ_{FC} – see Eq. (5)
5. The shape of the phase calibration – see Eq. (3) :

$$\varphi_{set,c}(\lambda) = \varphi_{set}(\lambda) + \mathrm{A}.\varphi_{set}(\lambda) + B.\varphi_{set}{}^2(\lambda) + C.\varphi_{set}{}^3(\lambda)$$

6. The shape of the converted laser spectrum: $S_c(\omega) = S(\omega) * |\mathcal{F}\{D\}|$

   D is the vector of 16 real numbers converted via Fourier transform $\mathcal{F}\{D\}$

Such merit function with many input parameters is expected to feature many local minima. Therefore, we took the fitting in several steps:

(i) Optimization of items 1 and 2 (5 parameters): we scanned the parameters across a reasonable range and identified the optimum value. We observed a single minimum, which was very strongly dependent only on two parameters: GDD and Δp

(ii) Optimization of items 1-5 (5 more parameters) using the previous optimum value as the starting point. We did not observe any significant shift of the previously fitted parameters from step (i).

(iii) Optimization of item 6 (16 parameters), where the optimized parameters from step (ii) are used as fixed values.

To verify convergence of the fitting, we modified and interchanged steps (ii)-(iii) to test the effect of the fitting procedure on the result. In general, we attained a different set of optimization parameters for different procedures. Nevertheless, the parameters with a significant effect on the pulse shape (GDD, Δp, σ_X) reached very similar values for all procedures. The resulting merit function values and G-errors were the same, irrespective of the fitting approach. We also tested the possibility to optimize each item 1-5 individually to get an improved agreement.

Naturally, the optimization of so many free parameters improved the agreement of the simulated and retrieved temporal shapes. The mean G_{sim} error was reduced in the best case two-fold compared to the initial value. However, even combining many correction factors did not provide a significantly better agreement between the six simulated and retrieved pulse waveforms. We

do not recommend fitting as the procedure to improve the model parameters. The fitting can be biased by the used set of phases, pulse retrieval imperfections and might be misleading for other pulses.

Instead, we propose it as a possibility to exclude the possibility that the pulse shaper simulation is exact, yet the input parameters suffered from uncertainty. In such a case, we would observe that the correction of the imprecise parameter would significantly affect the merit function value *M.*
